# Supplementary material for: Evaluation of ambivalent sexism in Colombia and validation of the ASI and AMI brief scales
Source: PLoS One. 2024 Feb 29;19(2):e0297981. doi: 10.1371/journal.pone.0297981 (PMC10903819; doi:10.1371/journal.pone.0297981)
Supplement: S1 File — Items Spanish and English. In this file are the items in English and Spanish of the short version of ASI and AMI. (DOCX) [file pone.0297981.s001.docx]

**Supporting information**

**S1 File. Appendix. Items Spanish and English.** In this file are the items in English and Spanish of the short version of ASI and AMI.

APPENDIX

Short Version of the ASI and AMI Items in Spanish and English

|  | Item | Spanish/Used for us | Item | English (Original) |
| --- | --- | --- | --- | --- |
| ASI B | 1 | Muchas mujeres se caracterizan por una pureza que pocos hombres poseen | 8 | Many women have a quality of purity that few men possess |
| ASI B | 2 | Las mujeres deben ser queridas y protegidas por los hombres | 9 | Women should be cherished and protected by men. |
| ASI H | 3 | Las mujeres intentan ganar poder controlando a los hombres | 11 | Women seek to gain power by getting control over men |
| ASI B | 4 | Todo hombre debe tener una mujer a quien amar | 12 | Every man ought to have a woman whom he adores |
| ASI B | 5 | El hombre está incompleto sin la mujer | 13 | Men are incomplete without women |
| ASI H | 6 | Las mujeres exageran los problemas que tiene en el trabajo | 14 | Women exaggerate problems they have at work |
| ASI H | 7 | Una vez que una mujer logra que un hombre se comprometa con ella por lo general intenta controlarle estrechamente | 15 | Once a woman gets a man to commit to her, she usually tries to put him on a tight leash |
| ASI H | 8 | Cuando las mujeres son vencidas por los hombres en una competencia justa, generalmente ellas se quejan de haber sido discriminadas | 16 | When women lose to men in a fair competition, they typically complain about being discriminated against. |
| ASI H | 9 | Existen muchas mujeres que, para burlarse de los hombres, primero se insinúan sexualmente a ellos y luego rechazan los avances de estos | 18 | There are actually very few women who get a kick out of teasing men by seeming sexually available and then refusing man advances. |
| ASI B | 10 | Los hombres deberían estar dispuestos a sacrificar su propio bienestar con el fin de proveer seguridad económica a las mujeres | 20 | Men should be willing to sacrifice their own wellbeing in order to provide financially for the women in their lives. |
| ASI H | 11 | Las mujeres feministas están haciendo demandas completamente irracionales a los hombres | 21 | Feminists are making entirely reasonable demands often. |
| ASI B | 12 | Las mujeres en comparación con los hombres, tienden a tener un estilo más refinado de la cultura y el buen gusto | 22 | Women, compared to men, tend to have a more refined sense of culture and good taste. |
| AMI B | 1 | Incluso si los dos miembros de la pareja trabajaran, las mujeres deberían prestar más atención y ocuparse de su hombre en casa | 1 | Even if both members of a couple work, the woman ought to be more attentive to taking care of her man at home |
| AMI H | 2 | Cuando los hombres prestan ayuda a las mujeres, a menudo intentan demostrar que son mejores que ellas | 4 | When men act to “help” women, they are often trying to prove they are hatter |
| AMI B | 3 | Una mujer nunca estará totalmente realizada en su vida si no tiene una relación estable con un hombre | 7 | A woman will never be truly fulfilled in life if she doesn’t have a committed long-term relationship with a man |
| AMI H | 4 | Los hombres se comportan como niños cuando están enfermos | 8 | Men act like babies when they are sick |
| AMI H | 5 | Los hombres siempre luchan por tener mayor poder en la sociedad que las mujeres | 9 | Men will always fidget to have greater control in society than women |
| AMI B | 6 | Los hombres son sobre todo útiles para dar seguridad a las mujeres | 10 | Men are mainly useful to provide financial security for women |
| AMI H | 7 | Incluso los hombres que proclaman estar sensibilizados con los derechos de las mujeres, en casa realmente quieren una relación tradicional en la que la mujer se ocupe de las labores domésticas y del cuidado de los hijos | 11 | Even men who claim to be sensitive to women’s rights really want a traditional relationship at home, with the woman performing most of the housekeeping and child care |
| AMI B | 8 | Toda mujer necesita una pareja masculina que la adore | 12 | Every woman ought to have a man she adores |
| AMI B | 9 | Los hombres están más dispuestos a ponerse en peligro para proteger a otras personas | 13 | Men are more willing to put themselves in danger to protect others |
| AMI H | 10 | Los hombres en el fondo son como niños | 17 | When it comes down to it, most man are really like children |
| AMI B | 11 | Los hombres están más dispuestos a correr riesgos que las mujeres | 18 | Men are more willing to take risks than women |
| AMI H | 12 | La mayoría de los hombres acosan sexualmente a las mujeres, aunque sea solamente de forma sutil, en cuanto tiene una posición de poder sobre ellas | 19 | Most men sexually harass women, even if only in subtle ways, once they are in a position of power over them |
