# Supplementary material for: Evaluation of ambivalent sexism in Colombia and validation of the ASI and AMI brief scales
Source: PLoS One. 2024 Feb 29;19(2):e0297981. doi: 10.1371/journal.pone.0297981 (PMC10903819; doi:10.1371/journal.pone.0297981)
Supplement: Attachment — Submitted filename: Response to Reviewers.docx [file pone.0297981.s002.docx]

Bogotá, November 4^th^ 2022

Response to Reviewers

Thanks for the suggestions and comments to improve the quality of our article. All comments were considered and the adjustment was realized.

**Reviewer #1**:

1. There are minor language editorial issues that should be looked into., maybe get a proofreader.

Authors comments: The document was completed reviewed and adjusted.

2. Page 3 has an author's name and year, is that the Vancouver excerption when it comes to discussing a theoretical framework?

Authors' comments: The mention of the Ambivalent Sexism Theory authors is important for the main idea of the paragraph. We adjusted the citation to the Vancouver format.

3. I suggest that the supporting information/appendices that appear at the end of the paper be moved either to the body of the text where there are discussions about the figures or before the bibliography so that the bibliography is the last section.

Authors' comments: We accept the suggestion. We included the Appendix before the bibliography.

4. I also suggest that for multiple sources which appear in-text, a comma is used to separate them instead of consecutive multiple brackets as seen for example at the top of page 18.

Authors' comments: We adjusted the multiple cites like the reviewer's recommendation.

5. If possible, include a conclusion heading.

Authors comments: We included the subtitle: “conclusion”.

**Reviewer #2:**

1. The title has been outlined together with the forms (HS and BS). Consider checking under the instruments heading that the ASI-brief and the AMI-brief share the same paragraphs.

Authors' comments: We reviewed and made them and made.

2. Too much spacing between the heading and subheading, consider removing the extra spaces and lines.

Authors' comments: We checked them and did them

Additional Adjusts:

1.

2. We checked them and did them.

3. We included a title page within our main document.

4.4 Unfunded studies: “The authors received no specific funding for this work.”

5.5 Competing Interests: “The authors have declared that no competing interests exist."

6.6 We included a title page within our main document.

7.7 Done

8.8 Done

I'd like to thank you for your valuable review.

Yours truly,

Lizeth Cristina Martínez-Baquero

PhD. Student

PhD. Pablo Vallejo Medina
