# Supplementary material for: Evaluation of ambivalent sexism in Colombia and validation of the ASI and AMI brief scales
Source: PLoS One. 2024 Feb 29;19(2):e0297981. doi: 10.1371/journal.pone.0297981 (PMC10903819; doi:10.1371/journal.pone.0297981)
Supplement: Attachment — Submitted filename: Reviewer_06_07_23.docx [file pone.0297981.s003.docx]

Reviewer Recommendation and Comments for Manuscript Number PONE-D-22-15490R1

**Materials and methods**

- The inclusion criteria of the participants are very generic
- Explain why there is a division of samples where a first group is subjected to an EFA and the second to a CFA.
- In the factors extracted in the EFA, they were obtained using 24 different methods, clarifying the relevance of these methods given the non-use of others valid for the EFA.

**Process**

- Specify the linguistic adjustment process for the two scales carried out.

**Discussion**

- A brief analysis of the results obtained by age, both in men and women, would have been very interesting.

**General features**

- There are very generic and absolute statements in the text, modify the wording of certain paragraphs such as:

“We found that women are more hostile and less benevolent towards men, indicating a negative evaluation and rejection of traditional gender relations in which women do not accept men's superiority in the non-domestic context”
