# Supplementary material for: Evaluation of ambivalent sexism in Colombia and validation of the ASI and AMI brief scales
Source: PLoS One. 2024 Feb 29;19(2):e0297981. doi: 10.1371/journal.pone.0297981 (PMC10903819; doi:10.1371/journal.pone.0297981)
Supplement: Attachment — Submitted filename: Review answer..docx [file pone.0297981.s004.docx]

Reviewer Recommendation and Comments for Manuscript Number PONE-D-22-15490R1

Thank you so much for the commentaries, they really improved the manuscript.

**Materials and methods**

- The inclusion criteria of the participants are very generic.

We have included more info.

- Explain why there is a division of samples where a first group is subjected to an EFA and the second to a CFA.

It is not recommended to confirm in a sample what has already been explored in that same sample. Logically, if we test the confirmation of the same model explored in the same sample, the fit will always be almost perfect. We have not added more information in this regard in the article.

-In the factors extracted in the EFA, they were obtained using 24 different methods, clarifying the relevance of these methods given the non-use of others valid for the EFA.

Sorry we test 14 no 24 methods. All of theme were adequate four the test/sample characteristics. Those were:

Acceleration factor

CNG

beta Multiple_regression

VSS complexity 1

VSS complexity 2

Velicer's MAP

Optimal coordinates

Parallel analysis

Kaiser criterion

t Multiple_regression

p Multiple_regression

Scree (R2)

Scree (SE)

BIC

BIC (adjusted)

Bentler

Bartlett

Anderson

Lawley

We have corrected the erratum and nuanced why we used them.

**Process**

- Specify the linguistic adjustment process for the two scales carried out.

In the cited paper (60) “Desarrollo de guías para adaptar cuestionarios dentro de una misma lengua en otra cultura” the details can be consulted in both English and Spanish.

**Discussion**

- A brief analysis of the results obtained by age, both in men and women, would have been very interesting.

This analysis is now included in the results section and briefly discussed in the discussion section.

**General features**

- There are very generic and absolute statements in the text, modify the wording of certain paragraphs such as:

“We found that women are more hostile and less benevolent towards men, indicating a negative evaluation and rejection of traditional gender relations in which women do not accept men's superiority in the non-domestic context”

We have modified the wording.
